# A woman living with osteoarthritis: A case report

**DOI:** 10.1186/1757-1626-1-153

**Published:** 2008-09-16

**Authors:** Jane C Richardson, Christian D Mallen, Helen S Burrell

**Affiliations:** 1 Arthritis Research Campaign National Primary Care Centre, Keele University, Keele, Staffs, ST5 5BG; 2 Kingsbridge Medical Practice, Kingsbridge Avenue, Clayton, Newcastle-under-Lyme, Staffordshire, ST5 3BR

## Abstract

Osteoarthritis is a common condition that is typically associated with older adults. Other causes of osteoarthritis, such as those cases resulting from childhood Perthes disease, can affect younger people and frequently have a major impact on the lives of those affected. This case report describes the experiences of one patient with osteoarthritis, using examples of her poetry to illustrate her social, psychological and emotional transformation.

## Introduction

Osteoarthritis (OA) is the most common joint disease and one of the most widespread of all chronic conditions managed in general practice. Whilst the prevalence of OA increases with age, a significant minority of adults experience symptoms earlier in life. Most cases of osteoarthritis are not extraordinary, yet the individual experiences of those affected provide a unique opportunity for health care practitioners and researchers to more fully understand the impact that common conditions have on their patients.

This case report was triggered by a patient (HB) wanting to tell her story. The importance of narrative in medicine is increasingly being recognised as a powerful tool that can strengthen clinical practice and help to create an alliance with patients. This alternative case report, written by a researcher (JCR), a general practitioner (CDM) and a patient (HB), presents a fairly typical case of a younger adult with osteoarthritis. However, rather than presenting laboratory results or X-ray findings, we use Helen's poems to highlight her experiences and her journey with osteoarthritis.

## Case presentation

Helen is 46 years old and has generalised osteoarthritis. Her hips and hands are the most severely affected joints. Helen's health problems started at the age of 11, following a fall on a cross-country run. She consulted her general practitioner and the accident unit multiple times with increasing levels of pain and disability before she was eventually diagnosed with Perthes disease, a diagnosis that has had a huge impact on her subsequent life. Over the years Helen has tried the full range of pain medications. A review of her past prescriptions reveals trials of over 15 different analgesics and anti-inflammatory drugs, covering the full range of the analgesic ladder. Her current medication, oxycodone, has so far been the best choice, optimising pain relief whilst minimising side effects. Helen continues to be under the care of local orthopaedic surgeons, who are contemplating further revisions to her failed total hip replacement. Helen remains independent and self-caring despite deterioration in her levels of pain and physical functioning.

Helen trained as a radiographer, but following the failure of a hip replacement she became a wheelchair user, which was not felt to be compatible with her job. This loss has had significant impact on Helen's identity: she describes it as being '*picked up from one life, where I could focus on me and what I wanted to happen, and put in another, where someone else was in control*'. The issues of identity and social roles are key for Helen, in knowing how to define herself – 'A*m I disabled? a woman? a disabled woman? a carer*?' – and in the huge loss of identity caused by loss of her job, associated financial security and her increasing disabilities. This meant radical changes to her social life, independence, choices, freedom of movement, interests and hobbies.

The nature of Helen's condition does not mean she has necessarily lost all other social roles. Helen's parents live close to her and suffer from a number of physical and mental health conditions. For Helen, being her parents' main carer is a source of positive identity and pride, although it also means she can be called to provide physical or emotional help at any time during the day or night. Her father's illness also meant that Helen took on some of his caring responsibilities, in looking after her elderly aunt who had dementia.

Relationships and friendships have been very important to Helen and have been affected by her condition. She had been engaged at age 21, shortly before a period of hospitalisation. The relationship ended, she felt, because of her depression during this period and her fiancé's lack of experience of dealing with such a situation. She describes the problems with actually meeting people with whom to have a relationship, but also the dilemma and difficulty of attempting to "*build an equal partnership, when somebody ****does ****have to take on a carer's role*." She also describes conflict in relationships with people who had had disabilities since birth, both from their perspective, and from expectations of other people who thought she 'should' have a relationship with a non-disabled person.

Helen describes the process of learning "*to become disabled*." An event she found helpful in this process was a disability awareness training course, because, "*you're put into this situation but you don't get given a tablet to make you be disabled, ...to understand being disabled and impaired and 'all the rest of it"." *This enabled her to write her own disability awareness course, including issues that were important to her as a woman and as a person who had acquired her disabilities.

Helen's relationship with her GP (CDM) is important to her, particularly as he is also her parents' GP and therefore has an understanding of the context in which she is managing her disability. She also feels strongly about the benefits of having a relationship with her GP in which she is seen as a person and is able to "*be herself*", without being concerned that she is seen as a problem or that people are worrying about her.

## Discussion

We have presented a case of a woman with osteoarthritis secondary to Perthes disease in childhood. We have described her emotional, social and psychological transformation using her poetry to illustrate different life experiences. The notion of biographical disruption[1] can be used to describe the identity changes experienced by Helen. This 'breaking down' of one's life is eloquently described by Arthur Frank, a medical sociologist who has himself experienced critical illness: "*What happens when my body breaks down happens not just to that body but also to my life, which is lived in that body. When the body breaks down, so does the life. Even when medicine can fix the body, that doesn't always put the life back together again*." [2] One response to biographical disruption is to attempt to repair the narrative of one's life[3]. Creativity, through poetry, writing or art, can be seen as one way of trying to make sense of this disruption. This type of creativity may be actively encouraged as part of a healing process [4-6], although Helen's poems were originally written solely for and by herself.

This is not a traditional case report, yet we believe that our approach can be equally as informative, by allowing doctors and researchers to more fully understand the impact a disease has on all aspects of their patient's lives.

## Patient's perspective

Where have I gone?

Where's the woman who weighed less than 9 stone?

Who wore a dress size 12 and didn't need to wear shapeless clothes or jogging suits?

Who had shaped and tidy eyebrows that would complement her latest hair colour and style?

Whose painted nails, with manicured hands and feet, were perfect for holidays in the sun?

Where's the woman who had a vocation not just a job, but who exists on benefits, a step away from poverty?

Where's the woman who owned her own home that gave her safety and privacy, it was her pride and joy?"

Where's the woman whose hobbies include travel, gardening, decorating, furniture restoring, sewing, reading and studying at home?

*Here I am and life before my impairment has gone, the only thing I can do is hold a pen with a special grip and writing is agony*.

Where has she gone?

Where has she gone?

WHERE HAVE I GONE?

*Do you see me?*

How can you ever know me, when all you see is my chair?

*My limitations are all you see and you say they complicate your life*.

Will you ever see the deep pools of love in my eyes for you?

*When you half close yours with pity and turn away from me*.

The beating of my heart in expectation of your closeness is

*quickly cooled by your fleeting hug or, worse, patting my shoulder*.

I wait in anticipation remembering the taste and softness of

*your kiss, you offer me a warm 'peck' on the cheek*.

I smell your aftershave ... you say I smell clean!

*I remember running my fingers through your hair*,

Ripping buttons off your shirt but that's difficult when you

*stand behind me pushing my chair, we can't even hold hands anymore*.

*The power of my emotions makes me feel strong*,

*Then I catch that pitying look in your eye*,

*They die in my heart*.

I do not speak, my smile fills my face but you will never know ...

You'll never see the real woman who is me

Who sits and is seen by the world framed by a wheelchair

*Cut off emotionally just because I cannot stand or walk*.

*Thank you*

Thank you to those who take the time to listen to difficult and unclear speech, for you help us to know that if we persevere we can be understood

Thank you to those who walk with us in public places, ignoring stares and whispers from strangers, for in your friendship we find enjoyment, laughter and happiness

Thank you for never asking us to 'hurry up', but even more special is you don't snatch our tasks from us or offer 'Care' in such a way as to make us feel that we are still children, with no control and respect

Thank you for standing beside us when we enter new experiences and try new adventures

Though our success may be outweighed by our failure, the experience will stay with us forever and there will be many occasions when we surprise ourselves and maybe even you!

*Thank you for asking for our help and expertise*,

As self-confidence and awareness come from being needed by you and others

Thank you for giving us respect

You acknowledge our value as experts in our fields and that we require to live with equality in society

We shouldn't have to ask or have laws to enforce it or remind you

*Thank you for assuring us that the things that make us individuals are not our medical impairments, as everyone has those and they don't define ONE'S SELF, it's people's attitudes that create barriers that exclude us from you*.

*Treat us as we treat you*.

## Consent

Written informed consent was obtained from the patient for publication of this case report and accompanying images. A copy of the written consent is available for review by the Editor-in-Chief of this journal.

## Competing interests

The authors declare that they have no competing interests.

## Authors' contributions

"JCR interviewed HSB and wrote the article based on the interviews. CDM wrote the medical aspects of the article and helped with the drafting of the article. HSB wrote the patient perspective section. HSB and CDM helped revise the manuscript".
